# Current Landscape of Generative Adversarial Networks for Facial Deidentification in Dermatology: Systematic Review and Evaluation

**DOI:** 10.2196/35497

**Published:** 2022-05-27

**Authors:** Christine Park, Hyeon Ki Jeong, Ricardo Henao, Meenal Kheterpal

**Affiliations:** 1 Department of Dermatology Duke University Medical Center Durham, NC United States; 2 Department of Biostatistics and Bioinformatics Duke University Durham, NC United States

**Keywords:** facial recognition, deidentification, facial photographs, HIPAA, dermatology, guidelines

## Abstract

**Background:**

Deidentifying facial images is critical for protecting patient anonymity in the era of increasing tools for automatic image analysis in dermatology.

**Objective:**

The aim of this paper was to review the current literature in the field of automatic facial deidentification algorithms.

**Methods:**

We conducted a systematic search using a combination of headings and keywords to encompass the concepts of facial deidentification and privacy preservation. The MEDLINE (via PubMed), Embase (via Elsevier), and Web of Science (via Clarivate) databases were queried from inception to May 1, 2021. Studies of incorrect design and outcomes were excluded during the screening and review process.

**Results:**

A total of 18 studies reporting on various methodologies of facial deidentification algorithms were included in the final review. The study methods were rated individually regarding their utility for use cases in dermatology pertaining to skin color and pigmentation preservation, texture preservation, data utility, and human detection. Most studies that were notable in the literature addressed feature preservation while sacrificing skin color and texture.

**Conclusions:**

Facial deidentification algorithms are sparse and inadequate for preserving both facial features and skin pigmentation and texture quality in facial photographs. A novel approach is needed to ensure greater patient anonymity, while increasing data access for automated image analysis in dermatology for improved patient care.

## Introduction

### Facial Deidentification in Dermatology

Over the last several years, there has been an explosion of artificial intelligence (AI) and deep learning for dermatological image analysis. These tools have demonstrated efficacy in the diagnosis and quantification of skin conditions at par with or surpassing human performance [1,2]. Additionally, there have been use cases in dermatology where the human eye is unable to precisely quantify the burden of disease, while AI can be used to support the clinical decision-making process with better consistency [3,4].

Facial image data are needed for developing models that evaluate attributes such as redness (ie, acne and rosacea models), texture (ie, wrinkles and aging models), pigmentation (ie, melasma, seborrheic keratoses, aging, and postinflammatory hyperpigmentation models), and skin lesions. To advance AI in dermatology, image data are needed at scale. For patient data to be used for research, consent may be obtained; however, for data at scale where this is not possible, adequate deidentification must be applied to images. Traditionally, journals have required facial feature concealment that typically covers the eyes, but these guidelines are largely insufficient to meet the ethical and legal guidelines from the Health Insurance Portability and Accountability Act for patient privacy and identity protection [5,6]. Facial features, tattoos, jewelry, birthmarks, and other identity-informative background features are additional features that are considered identifying; facial feature deidentification is considered the most challenging task, given a lack of expert consensus and a lack of testing infrastructure and quantitative metrics for adequacy of automatic and manual facial image deidentification algorithms.

Identity protection challenges extend to other industries involved with facial images as well as video privacy. Hence, there have been increasing efforts to propose facial deidentification algorithms in the literature with corresponding use cases. Ideally, the methods should both hide the original identity of participants and preserve data reusability. We hypothesize that automated facial deidentification algorithms currently proposed in the literature may be useful for dermatological research use. To this end, we conducted a systematic review to search for studies reporting facial deidentification and summarized their proposed methodology and application to image analysis in dermatology.

### Comparison of Different Facial Deidentification Algorithms

Conventional methods of ad hoc facial deidentification use blur [7], pixelation [8], masking, random swapping, perturbation, and face region replacement [7,9-18] to obfuscate parts or entire images to protect visual privacy. This set of obfuscating techniques prevent the rendering of the original image, but they do not necessarily guarantee preservation of privacy (ie, masks and blur can be removed) and often compromise data utility (ie, preservation of dermatological characteristics with diagnostic value) [19,20]. To test if these techniques protect privacy, studies have explored whether these methods can fool computer and human detection. Many studies have successfully avoided detection by use of computer algorithms but have found that human eyes can easily notice the alteration [21-24]. Furthermore, simply applying distorting filters to images risks identity revelation after reconstruction [13].

The *k*-anonymity–based algorithms were proposed as one of the original feasible approaches in solving this issue of data utility after deidentification [25]. Briefly, the *k*-anonymity–based methods and their variations deidentify an image by replacing the face with the average of *k* images from a given collection of images, and they achieve privacy protection with a rate lower than 1/*k*. The most commonly used *k*-algorithm is from the *k*-Same family [8,13,17]. However, one of the key issues with the variations of the *k*-Same algorithm is the introduction of ghosting artifacts caused by the misalignment of images. The ghosting artifacts compromise privacy protection by making the images appear unnatural. The ghosting effect can be overcome by employing a large *k* in the algorithms, but this requires a large image collection, otherwise it results in a lack of distinction among the deidentified faces; this is because the number of discriminative faces in the deidentified face set is limited by the total number of images divided by *k*. This is problematic for applications in skin image analysis in dermatology because adequate privacy protection is achieved with averaging a greater number of images, which, in turn, will dilute redness, pigmentation, and other image attributes that are critical to dermatologic data utility. In other words, there is an intrinsic trade-off when choosing *k* between identifiability and preservation of dermatological features.

The *k*-Same-M algorithm was developed to eliminate the ghosting effects in order to enhance privacy protection with minimal loss of data utility [26]. This algorithm uses an active appearance model (AAM), which is an algorithm that can reconstruct an image representation based on its shape and texture [26]. In this way, an AAM coupled with the *k*-based algorithms can help reduce the ghosting effect in the deidentified images by ensuring a better alignment of the synthesized identity onto the original images. However, the reconstructed images from an AAM are still averaged images from the respective data set and, hence, some important aspect of data utility, such as facial expression, could be compromised.

Another technique for achieving facial deidentification is through the use of machine learning methods involving deep neural networks [27-31]. Convolutional neural networks (CNNs) are effective in extracting features from raw faces and, hence, facilitate image transformation into target outcomes. Limitations associated with methods involving CNNs and convolutional autoencoders are that they are time costly because they require a large sample size to be trained and optimized. Specifically for CNNs, these are supervised algorithms that also need labels for ground-truth classifications. Furthermore, the output images are still not natural enough to effectively preserve privacy.

Generative neural networks (GNNs) constitute a novel method to generate realistic face surrogates that can be used for deidentification. This quality can be exploited to retain skin attribute quality from a source image of interest. These also allow for retaining certain aspects of the original data, such as age, gender, and facial expressions, while replacing sensitive personal attributes with artificial objects, such as facial features. GNNs are originally based on generative adversarial networks (GAN), which combine a generative model that produces a synthetic image and a discriminator (ie, critic) network that classifies the synthetic image as either real or artificial. This method works by training the discriminator network as a standard classifier to distinguish between the two image sources as real or artificial and training the generative network as an image-generating model that can fool the discriminator network, with the goal of generating the most realistic-appearing synthetic images [32]. The model is improved in an adversarial manner via back-propagation with both generative and discriminator networks to identify the generator’s parameters that should be optimized to make the generated images increasingly challenging for the discriminator. After completion of training, the output images from the generator network should be indistinguishable from the real images for the discriminator as well as look visually convincing for humans [13,25,33-35].

The use of GANs in facial deidentification is intriguing due to their potential for disentanglement of facial features and skin attributes. Theoretically, facial images can be deidentified by a GAN that recognizes facial features, such as eyes, nose, and lips, and then replaces them with features from another facial image, while continuing to preserve the realistic-appearing facial image as well as features of interest, such as redness, pigmentation, texture, and skin lesions. Hence, based on their high data utility, GANs hold the promise of privacy protection by completely changing image identification by human and automated detection. This study focused on reviewing the GAN-based models published to date for facial deidentification for dermatologic use cases. We also evaluated the performance of top-performing GANs in deidentifying dermatological images while preserving the important facial and skin quality features in these images.

## Methods

### Search Strategy

We conducted a systematic search using a combination of headings and keywords to encompass the concepts of facial deidentification and privacy preservation. The MEDLINE (via PubMed), Embase (via Elsevier), and Web of Science (via Clarivate) databases were queried from inception to May 1, 2021. We also performed referential backtracking on the most recent studies to ensure inclusion of all relevant articles. Studies of incorrect design and outcomes were excluded during the screening and review process. The search strategies are outlined in Multimedia Appendix 1.

### Definitions and Inclusion and Exclusion Criteria

Facial features were defined as identifying features associated with an individual, including the eyebrows, eyes, nose, mouth, and ears. For deidentification in dermatologic use cases, these features are important to remove and replace. The skin was then defined as the remaining facial area bounded by the hairline. Preservation of skin quality by algorithms was evaluated as to how well the algorithms preserved the quality of the skin tone and texture from the input images. We included studies that focused on variations of the GAN algorithm for the purpose of facial deidentification in images, video, or both. Studies were excluded if they focused on any other facial deidentification algorithms due to low preservation of pixel-level skin quality based on the methodology.

### Ethics Approval

This study was approved by the Institutional Review Board (Retrospective cutaneous dermato-oncological conditions treated by dermatology service) for protocol No. Pro00100765. Patient consent was not required due to the nature of this study.

## Results

### Overview

**Figure 1:**
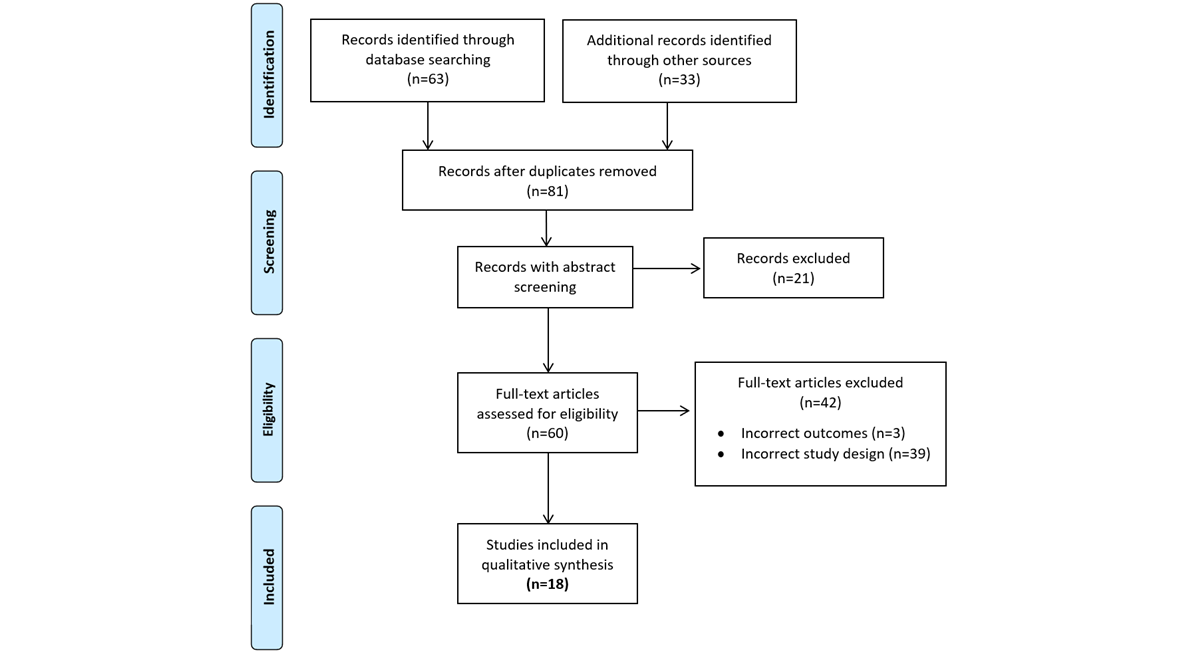
PRISMA (Preferred Reporting Items for Systematic Reviews and Meta-Analyses) diagram.

A total of 18 studies using GAN methodology were included in the final review (Figure 1). Table 1 [36-53] summarizes the different types of GAN algorithms and the goals of all the studies as well as an evaluation of their ability to preserve skin quality (ie, color and texture), capacity for data utility, and demonstration of adequate facial deidentification with human eyes based on the results illustrated in the studies. We then applied two of the best GAN-based algorithms that were publicly available to the SD-260 (260 classes of skin diseases) data set [54], a public data set of images of dermatological conditions, to assess whether the output images appropriately preserved skin quality.

**Table 1:** Overview of included GAN-based studies.

Author, year	Method of facial deidentification	Novelty in proposed method of facial deidentification	Skin attribute preservation				Data utility		Facial deidentification (human)
			Color	Texture					
Pan et al, 2019 [36]	*k*-Same-Siamese-GAN^a^	Maintenance of high resolution of images to preserve their utility	Partial		No	Low		Yes	
Song et al, 2019 [37]	Evolutionary GAN	Structural similarity index and the distance between the original face and the deidentified face	Partial		Partial	Low		No	
Agarwal et al, 2021 [38]	StyleGAN and GAN	Preservation of emotion and nonbiometric facial attributes of a target face	N/A^b^		No	Low		Yes	
Nitzan et al, 2020 [39]	Disentanglement coupled with GAN	Disentanglement of identity from other facial attributes with minimal training	Yes		No	High		No	
Lin et al, 2021 [40]	Facial privacy GAN for social robots	Strengthened feature-extraction ability to improve the discriminatory accuracy	Partial		No	Low		Partial	
Maximov et al, 2020 [41]	Conditional identity anonymization GAN	Development of a model for image and video anonymization with removal of identifying characteristics of faces and bodies	Yes		No	High		Yes	
Brkic et al, 2017 [42]	Conditional GAN	Production of realistic deidentified human images that avoid human- and machine-based recognition	N/A		N/A	Low		N/A	
Meden et al, 2017 [43]	Generative neural network	Synthesis of artificial surrogate faces with preservation of nonidentity-related aspects of the data for data use	No		No	Low		Yes	
Mirjalili et al, 2017 [44]	Convolutional autoencoder using semiadversarial network	Autoencoder-based transformation of an input face image	N/A		No	Low		No	
Radford et al, 2016 [45]	DCGAN^c^	Unsupervised GAN	No		No	Low		No	
Wu et al, 2019 [46]	Privacy-protective GAN	Privacy protection, utility preservation, and structure similarity	N/A		Partial	Low		Yes	
Hukkelås et al, 2019 [47]	Conditional GAN	Novel generator architecture for face anonymization via synthesis of realistic faces	No		No	Low		Yes	
Ren et al, 2018 [48]	Multitask extension of GAN	Deidentification in video with preservation of action	No		No	High		Yes	
Sun et al, 2018 [49]	DCGAN	Novel head inpainting obfuscation technique	Partial		No	Low		Yes	
Sun et al, 2018 [50]	GAN	New hybrid approach for identity obfuscation in photos via head replacement	Partial		No	Low		Yes	
Bao et al, 2018 [51]	GAN	Disentanglement of identity and attributes from faces for recombination into different identities and attributes for identity-preserving face synthesis in open domains	No		No	High		No	
Li et al, 2019 [52]	Adaptive embedding integration network	High-fidelity face swapping	Yes		No	High		Yes	
Nirkin et al, 2019 [53]	Face-swapping GAN	Face re-enactment with adjustment for pose and expression variations	No		No	High		Yes	

^a^GAN: generative adversarial network.

^b^N/A: not applicable; this information was not reported in this study.

^c^DCGAN: deep convolutional generative adversarial network.

### Disentanglement-Coupled GAN

**Figure 2:**
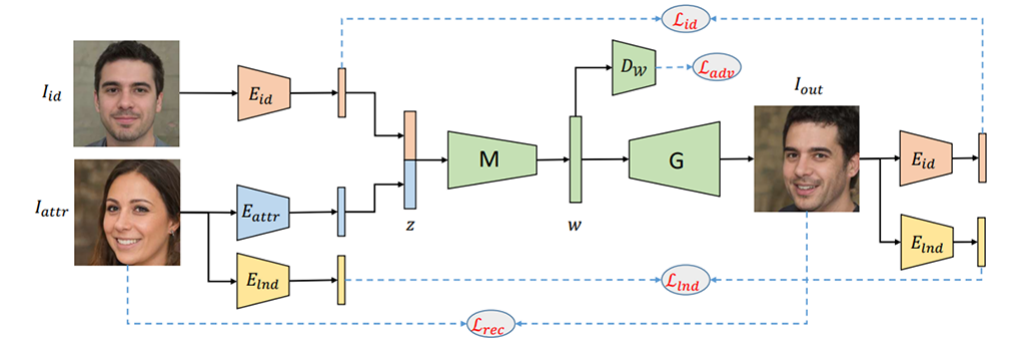
Disentanglement scheme. Solid lines indicate data flow and dashed lines indicate data loss. The identity and attribute codes are first extracted from two input images using encoders *E_id_* and *E_attr_*, respectively. Through the mapping network *M*, the concatenated codes are mapped to *W*, the latent space of the pretrained generator *G*, which, in turn, generates the resulting image. An adversarial loss *L_adv_* ensures proper mapping to the *W* space. Identity preservation is encouraged using *L_id_*, which penalizes differences in identity between *I_id_* and *I_out_*. Attribute preservation is encouraged using *L_rec_* and *L_lnd_*, which penalize pixel-level and facial landmark differences, respectively, between *I_attr_* and *I_out_* (reproduced from Nitzan et al [39], with permission from Yotam Nitzan). D_w_: discriminator; Elnd: landmark encoder; z: latent code.

One of the algorithms we chose was the disentanglement-coupled GAN presented by Nitzan et al [39]. The goal of this model is to generate an image by combining the identity of a given identity image with the attributes extracted from an attribute image. The author generates 70,000 images using StyleGAN [55], which are then used as the training data set. Identity is preserved by penalizing the identity difference between the identity image and attribute image. Attribute preservation is achieved by penalizing the difference in pixel-level and facial landmarks between identity image and attribute image. The network architecture is illustrated in Figure 2.

The performance of this method was compared against previously published methods, such as latent optimization for representation disentanglement [56], FaceShifter [52], and face-swapping GAN [53], for qualitative assessment; the performance was also compared against the adversarial latent autoencoder (ALAE) method [57] and the pixel2style2pixel (pSp) method [58] for quantitative assessment. Qualitatively, the authors demonstrated that their method showed better preservation for facial expression (ie, attribute image), head shape, and hair (ie, identity image) compared to the other models noted above. Quantitatively, the reconstruction performance was assessed by measuring pixel-wise reconstruction and preservation of semantic features, followed by comparison of the outcome to that of ALAE and pSp methods. This evaluation indicated that the pSp method showed better performance, but the author emphasized that their method was mainly for disentanglement and was not necessarily designed to reconstruct pixel-level information for reconstruction. This indicates that the model was able to replace and preserve realistic facial features, head shape, hair, and expressions due to superior performance of the disentanglement component while compromising pixel-level detail.

**Figure 3:**
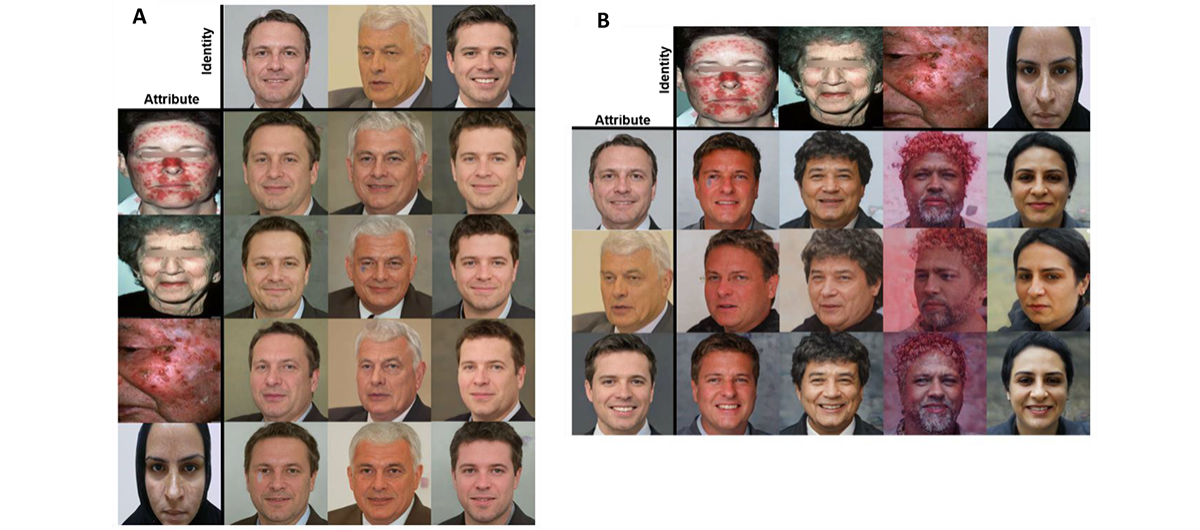
Output using the disentanglement-coupled GAN on dermatological images derived from the SD-260 data set. (A) Identity images assuming the facial pose and alteration of facial features from the attribute images. The attribute images fail to transfer the features of interest (ie, redness and pigmentation). (B) When switching the identity images to the images with features of interest, the model fails to preserve the dermatological features. GAN: generative adversarial network; SD-260: 260 classes of skin diseases.

When applying the disentanglement-coupled GAN to the SD-260 data set, there were two sources for the input data: one for *identity* and another for *attribute*. For this model, we experimented with whether the attributes, such as redness and pigmentation, of the faces from the dermatological images could be encoded in a new identity. Figure 3A shows the qualitative results derived from the model: in the data set where the images of interest, with redness and pigmentation, are the *attribute* images, there is no transfer of skin features of interest, only transfer of facial positions and expressions. Figure 3B shows that when the images of interest are the *identity* images, features are transferred without pixel-level accuracy to preserve high data utility for dermatology use. Overall, we can see that while the model generates realistic faces, it is unable to preserve pixel-level details of the faces.

### Conditional Identity Anonymization GAN

**Figure 4:**
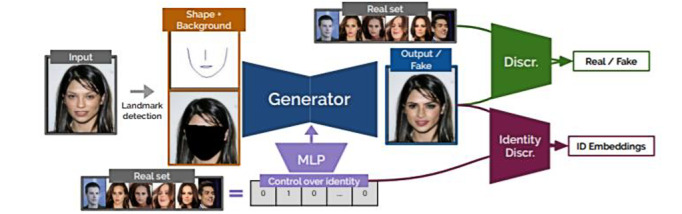
CIAGAN model scheme. The model takes the image and its landmarks, the masked face, and the desired identity as input. The generator is an encoder-decoder model where the encoder embeds the image information into a low-dimensional space. The identity given as a one-hot label is encoded via a transposed convolutional neural network and is fed into the bottleneck of the generator. Then, the decoder decodes the combined information of source images and the identities into a generated image. The generator plays an adversarial game with a discriminator in a standard GAN setting. Finally, the identity discriminator network is introduced, whose goal is to provide a guiding signal to the generator about the desired identity of the generated face (reproduced from Maximov et al [41], with permission from Laura Leal-Taixe). CIAGAN: conditional identity anonymization generative adversarial network; GAN: generative adversarial network.

The goal of this paper was to develop a model that can deidentify images and videos while preserving features for other computer vision tasks, such as detection, tracking, or recognition [41]. The overview of the methodology is as follows. The method first extracted the landmarks of a given image that contained a sparse representation of the face with limited information on the identity. This allowed the generator to adjust to the face shape, which enabled better preservation of the input pose. The authors used only the face silhouette, the mouth, and the bridge of the nose instead of using all 68 landmarks in order to allow the network to freely choose the facial features. The method also extracted masked background images to allow the model to learn to generate faces and not the background. Once the landmark and the background were extracted, the method used a conditional GAN (CGAN) [59] to generate realistic images by encoding the landmark and masked image and combining them with the identity images to feed into the decoder. The generated output image was then fed into the identity discriminator network to prevent the network from generating faces similar to the training data set and to ensure facial anonymization. The model architecture is shown in Figure 4.

The model was trained and evaluated on three public data sets: CelebA (CelebFaces Attributes), MOTS (Multi-Object Tracking and Segmentation), and Labeled Faces in the Wild. The performance of the model was assessed by using face detection and reidentification metrics with other existing methods, such as blurring and pixelization. When compared with a state-of-the-art facial deidentification method by Gafni et al [60], conditional identity anonymization GAN (CIAGAN) showed better deidentification rates by computer detection on two different data sets. The authors concluded that their method can both deidentify the source images better and generate much more diverse images compared to Gafni et al’s method.

**Figure 5:**
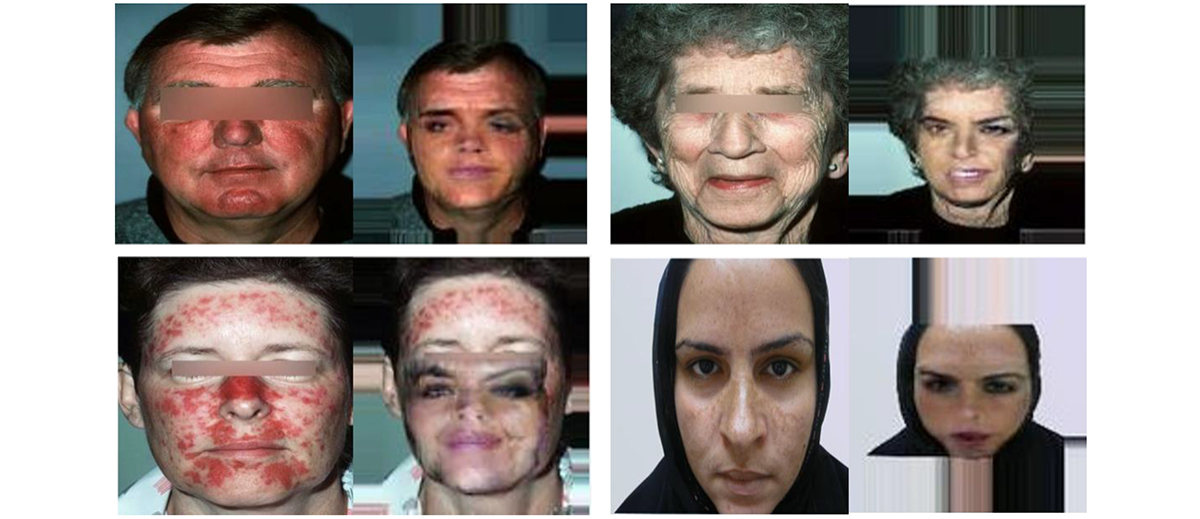
Output using CIAGAN on dermatological images derived from the SD-260 data set. Images on the left serve as source images, and a facial swap is done on the mid and lower part of the face for the images on the right. Generated images are of poor quality and only partially preserve facial attributes. CIAGAN: conditional identity anonymization generative adversarial network; SD-260: 260 classes of skin diseases.

When we applied the CIAGAN to the SD-260 data set, we first processed the landmarks of the dermatological images. Then, we allowed the model to deidentify each individual’s face from the processed landmark and background images. The model was pretrained using 1200 identities from the CelebA data set. Figure 5 shows the result from this model. The qualitative results show a reduction in pixel-level resolution as well as poor preservation of the dermatological attributes of interest in the mid to lower part of the face, while preserving the skin features of interest (ie, redness and pigmentation) in the forehead area. While this is a good method for facial swapping, CGAN at this level fails to preserve significant areas of interest with high-utility pixel-level detail.

## Discussion

### Principal Findings

Apart from the conventional facial deidentification methods, many of the advanced algorithms aim to preserve key facial features and expressions while maintaining privacy protection for the input images. Specifically, for GANs, there exist three major general limitations with these algorithms. Firstly, the outputs from these models that use face synthesis exhibit significant similarities between the synthetic and original images [61], which can be detected via human evaluation. Many of the currently existing algorithms are effective at modifying the images to avoid identification by face recognition software [17] but are not good enough to pass deidentification by humans. Thus, additional effort needs to be focused on addressing human detection, such as facial feature swap. Secondly, it is difficult to integrate the synthesized faces smoothly into the original image and make the images look unnatural, which compromises privacy protection [17,62]. Finally, synthetic faces can decrease data usability due to changes in skin attributes, such tone and texture, and due to changes in patient identity, such as age, gender, and race [13,49,63-65]. Particularly for medical applications, even with the recently developed, well-intentioned algorithms, such as disentanglement and CIAGAN, the existing facial deidentification models fail to precisely and accurately preserve the color and texture of the facial skin for applications in their attempt to protect the identity of individuals with dermatological conditions, such as rosacea, melasma, among others, included in the data sets. Hence, the challenge involved with sharing large data sets that include facial images of patients with dermatological conditions, while adequately protecting their identity, remains unresolved.

The current standards for deidentifying patient images involve blurring, pixelating, and masking out important identifying facial features, such as the eyes and eyebrows [6]. Kuang et al [66] showed that pixelation and blurring demonstrate high deidentification performance on computer detection compared to other advanced methods, such as privacy-protective GAN [67], natural and effective obfuscation [49], and AnonymousNet [63], which is one of the reasons that they remain as popular methods of facial deidentification. However, these conventional methods are at risk of identity restoration via decoding and reconstruction.

We propose that an ideal facial deidentification algorithm for dermatological application needs to (1) preserve facial architectural (ie, shape and gender) and skin features (ie, color and texture) to maintain data utility, while achieving adequate deidentification, and (2) avoid detection by computer and human analysis. To optimally protect the privacy of individuals in the images, the algorithm must be able to modify the image in a way that will be perceived as unaltered. In other words, the replacement identity will need to fuse well with the original content of the image. However, while altering the original content of the image, the skin attributes have to be preserved well enough so that the data utility of the data set involving the dermatological condition is not lost.

Herein, we demonstrate the utility of GAN-based facial deidentification methods to serve as use cases for AI development in dermatology, such as models quantifying redness (acne, rosacea, dermatitis, etc), pigmentation (melasma, postinflammatory hyperpigmentation, lentigines, etc), and texture (aging-related changes, volumetric assessment for neurotoxins or fillers, etc). While GAN development efforts for facial deidentification are not currently focused on skin-based use cases, focusing future efforts to achieve these goals can lead to an optimal facial deidentification model for dermatology.

### Conclusions

Although facial deidentification is a rapidly evolving field with several advanced algorithms for achieving facial deidentification by computer-level recognition, their application to dermatology use cases is currently suboptimal. However, GAN-based models have the potential to preserve skin attributes while replacing facial features that risk detection, holding promise to solve the dilemma of data sharing while preserving patient privacy and identity. Future work should focus on developing a model that can achieve both skin attribute preservation as well as detection avoidance by both computers and humans.

## Appendix

Multimedia Appendix 1 Search strategy.

## Abbreviations

AAM: active appearance model
AI: artificial intelligence
ALAE: adversarial latent autoencoder
CelebA: CelebFaces Attributes
CGAN: conditional generative adversarial network
CIAGAN: conditional identity anonymization generative adversarial network
CNN: convolutional neural network
GAN: generative adversarial network
GNN: generative neural network
LORD: latent optimization for representation disentanglement
MOTS: Multi-Object Tracking and Segmentation
pSp: pixel2style2pixel
SD-260: 260 classes of skin diseases
