# Supplementary material for: Current Landscape of Generative Adversarial Networks for Facial Deidentification in Dermatology: Systematic Review and Evaluation
Source: JMIR Dermatol. 2022 May 27;5(2):e35497. doi: 10.2196/35497 (PMC10334885; doi:10.2196/35497)
Supplement: Multimedia Appendix 1 [file derma_v5i2e35497_app1.pdf]

Multimedia Appendix 1. Search strategy

1. MEDLINE (via PubMed)
((de-identification) AND (facial)) AND (privacy)
Search: **((de-identification) AND (facial)) AND (privacy)** Sort by: **Most Recent**
("data anonymization"[MeSH Terms] OR ("data"[All Fields] AND "anonymization"[All Fields]) OR "data anonymization"[All Fields] OR ("de"[All Fields] AND "identification"[All Fields]) OR "de identification"[All Fields]) AND ("face"[MeSH Terms] OR "face"[All Fields] OR "facial"[All Fields] OR "facials"[All Fields]) AND ("privacies"[All Fields] OR "privacy"[MeSH Terms] OR "privacy"[All Fields])
Translations
de-identification: "data anonymization"[MeSH Terms] OR ("data"[All Fields] AND "anonymization"[All Fields]) OR "data anonymization"[All Fields] OR ("de"[All Fields] AND "identification"[All Fields]) OR "de identification"[All Fields]
facial: "face"[MeSH Terms] OR "face"[All Fields] OR "facial"[All Fields] OR "facials"[All Fields]
privacy: "privacies"[All Fields] OR "privacy"[MeSH Terms] OR "privacy"[All Fields]

2. Embase (via Elsevier)
Sources: Embase, Embase Classic, MEDLINE
Query: 'privacy' AND 'de identification' AND facial
Mapped terms: n/a

3. Web of Science (via Clarivate)
TOPIC: (de-identification) *AND* **TOPIC:** (facial)
Timespan: All years. **Indexes:** SCI-EXPANDED, SSCI, A&HCI, CPCI-S, CPCI-SSH, BKCI-S, BKCI-SSH, ESCI, CCR-EXPANDED, IC
